# Supplementary material for: CryoEM structure and assembly mechanism of a bacterial virus genome gatekeeper
Source: Nat Commun. 2022 Nov 26;13:7283. doi: 10.1038/s41467-022-34999-8 (PMC9701221; doi:10.1038/s41467-022-34999-8)
Supplement: Supplementary file 1 — Supplementary Information [file 41467_2022_34999_MOESM1_ESM.pdf]

CryoEM structure and assembly mechanism of a bacterial virus genome gatekeeper

Igor Orlov[1,2]*, Stéphane Roche[3]*, Sandrine Brasilès[3], Natalya Lukoyanova[4], Marie-Christine Vaney[5], Paulo Tavares[¶,3], Elena V Orlova[¶,4]

[1]Centre for Integrative Biology (CBI), Department of Integrated Structural Biology, IGBMC, Université de Strasbourg, 67404 Illkirch, France
[2]Current address: University of Glasgow, Scottish Centre for Macromolecular Imaging, Sir Michael Stoker Building, 464 Bearsden Road, Glasgow, G61 1QH, Scotland, UK
[3]Université Paris-Saclay, CEA, CNRS, Institute for Integrative Biology of the Cell (I2BC), 91198, Gif-sur-Yvette, France
[4]Institute of Structural and Molecular Biology, Department of Biological Sciences, Birkbeck College, Malet Street, London, WC1E 7HX, UK
[5]Institut Pasteur, Université Paris Cité, CNRS UMR3569, Unité de Virologie Structurale, 75015 Paris, France

*Equal contribution

¶ Corresponding authors:
Elena V. Orlova, e-mail: e.orlova@bbk.ac.uk
Paulo Tavares, e-mail: paulo.tavares@i2bc.paris-saclay.fr

Supplementary information

Supplementary Figs. 1 to 8
Supplementary Tables 1 to 4
Supplementary References

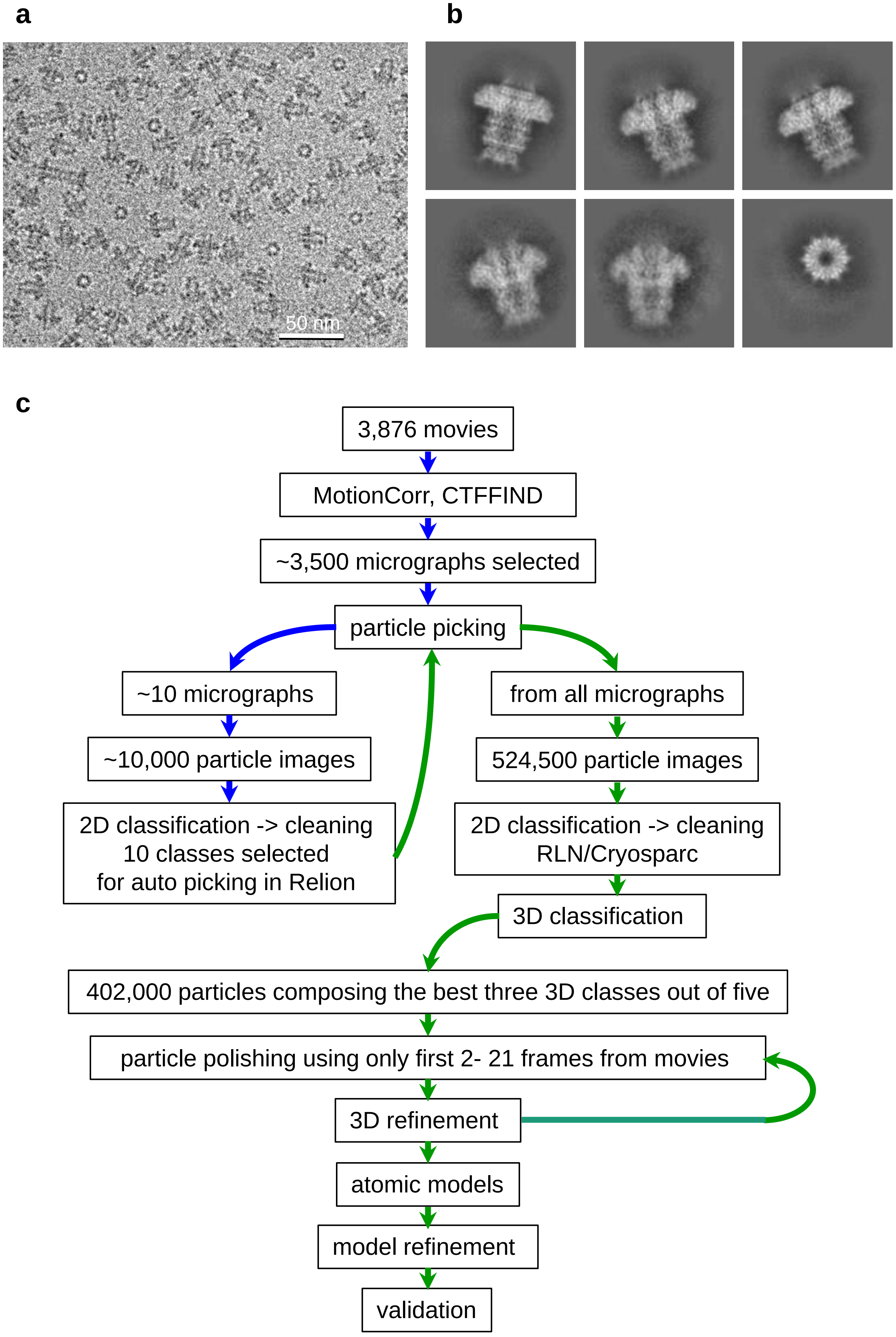

Supplementary Fig 1. Overall diagram of the image processing protocol.

Supplementary Fig 1. Overall diagram of the image processing protocol. a. A representative cryo-EM micrograph of the SPP1 connector. **b**. representative 2D classes of cryo-EM images of the connector (side views and one end view). **c**. The workflow of the cryo-EM image analysis, blue arrows show the link of the first steps from data collection to 2D classification and initial reconstruction. Numbers of micrographs and particle images used during processing are indicated in the corresponding boxes. The green arrows show the workflow of the entire data set processing. The refined map of the connector complex was calculated using ~402,000 particle images.

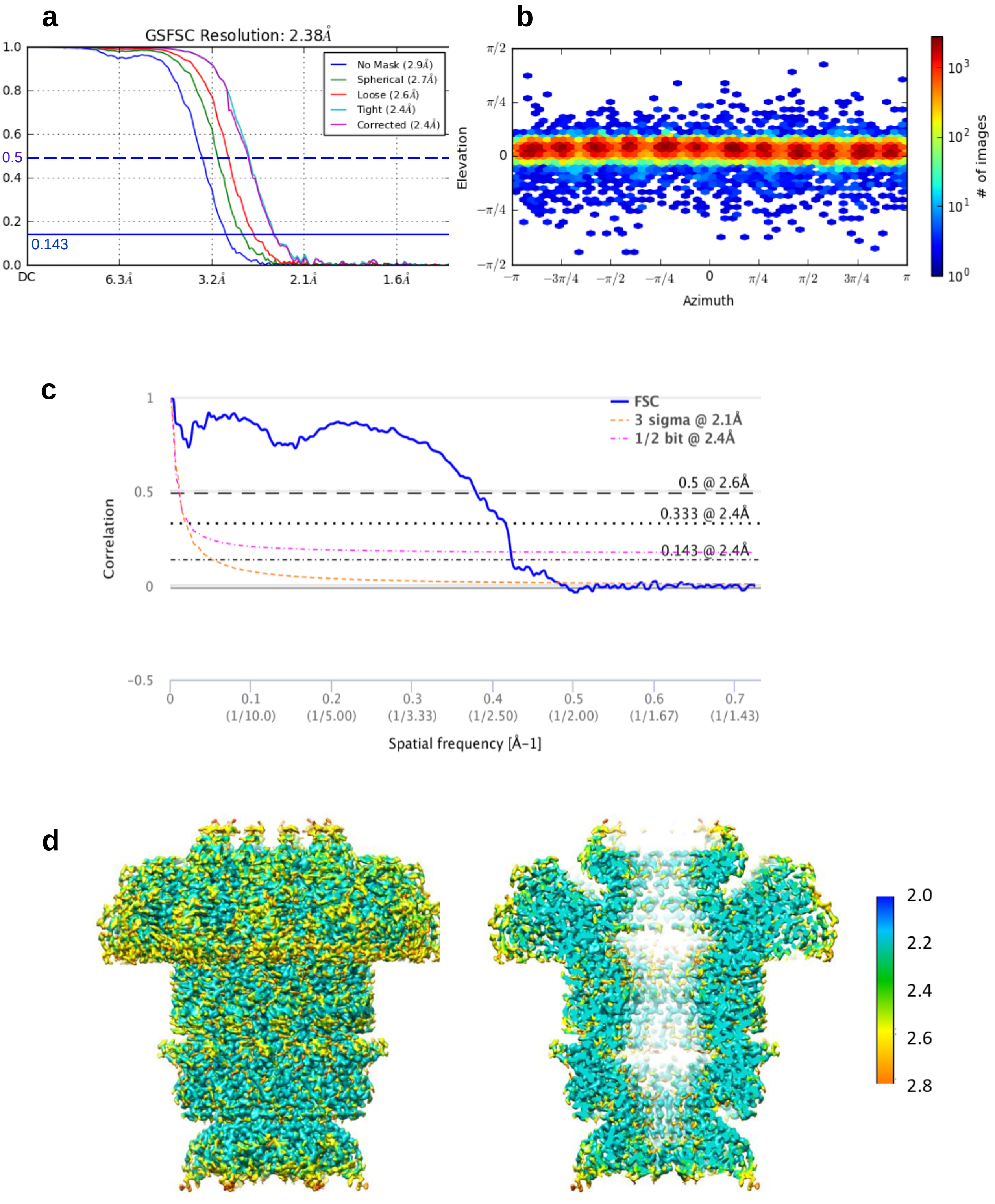

Supplementary Fig 2. Assessment of the map resolution.

e

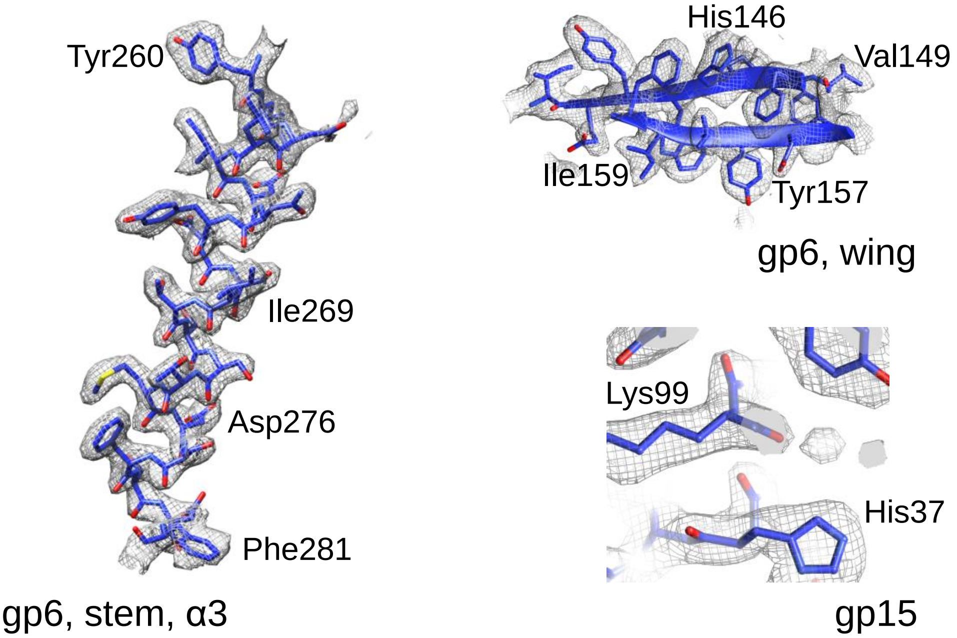

Supplementary Fig 2. Assessment of the map resolution.
a. Fourier shell correlation (FSC) curves for the SPP1 connector map. The FSC curves are calculated using different types of masks: the curve in blue is the FSC between two cryo-EM maps without any mask; the curve in green with a spherical mask; the curve in red with a loose mask; the curves in purple and cyan are FSCs between two cryo-EM maps using tight masks, and the fitted, generated, atomic model. At the threshold of 0.143 resolution was 2.38 Å, at the threshold of 0.5 it was 2.7 Å. **b.** Angular distribution of particle images used at the reconstruction. The colours indicate the number of images that have such orientations according to the bar shown on the right. **c.** Fourier shell correlation (FSC) between SPP1 connector map and fitted atomic models of three components of the connector gp6, gp15, and gp16. **d.** The EM map of the SPP1 connector coloured according to local resolution. The middle panel displays a cut-away view of the map allowing to see the details and resolution within the connector channel. Regions at higher resolution are in cyan, regions at lower resolution are in orange. Colour bar is shown in the right panel**.** **e.** Examples of fitting of the atomic models into EM densities. The left panel shows fitted helix α3 of the gp6 stem domain with side chains into the EM density (grey mesh). A fitted β-hairpin of the wing domain is shown in the upper right panel. A region of gp15 with fitted residues is shown in the bottom right panel.

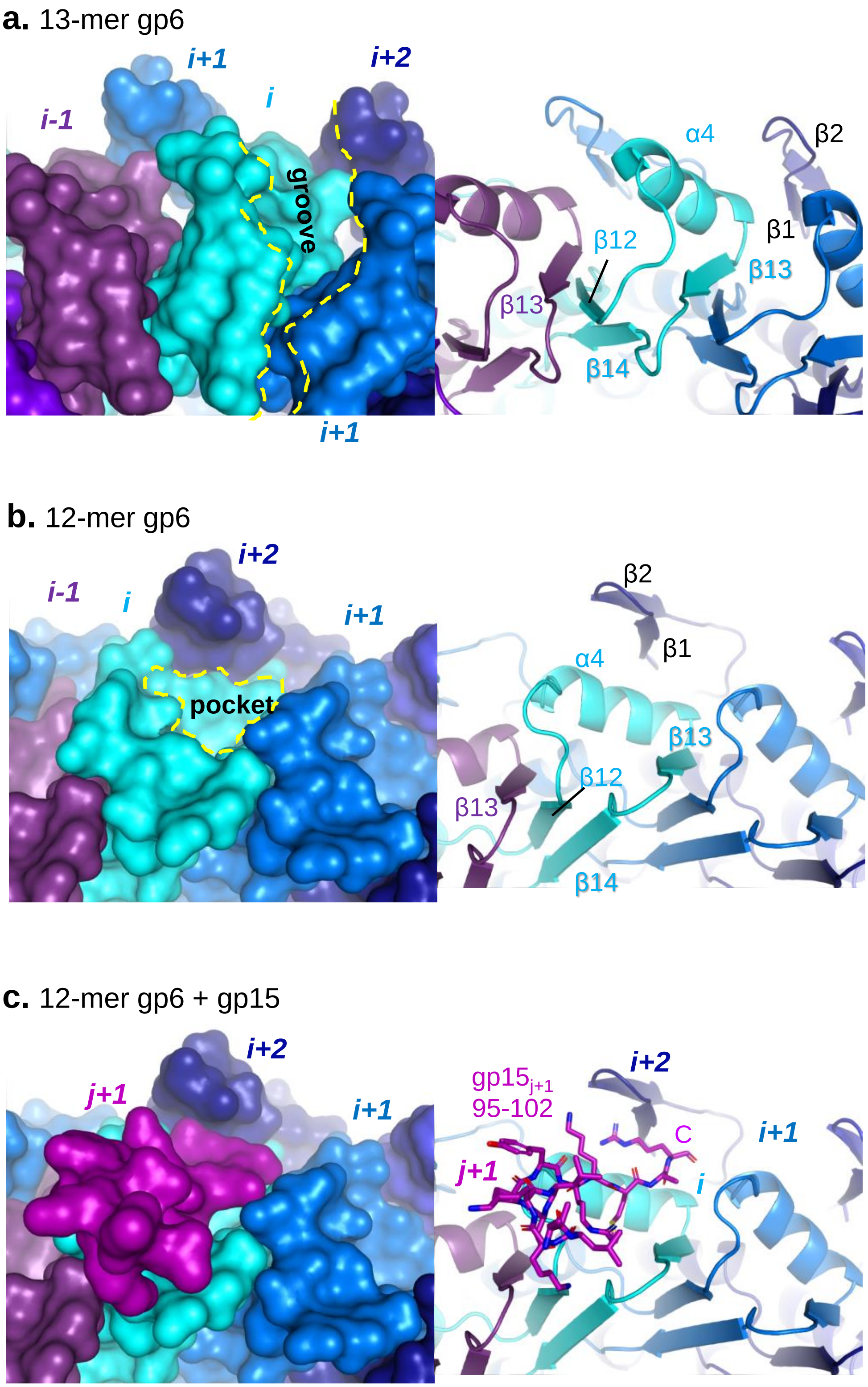

Supplementary Fig 3. The binding interface of gp6 clip domain and gp15.

Supplementary Fig 3. The binding interface of gp6 clip domain and gp15. a. Surface (left) and cartoon (right) views of three gp6 subunits in the assembly-naïve 13-mer. Secondary structure elements are labelled on the right panel and subunits are color-coded as in Fig. 1b. The 13-mer inter-subunit interface forms a groove in the clip (outlined by dashed curve in yellow). **b.** The pocket cavity is formed by three adjacent gp6 subunits *i, i+1*, and *i+2.* The area of interactions between the connector gp6 12-mer and gp15 is outlined by dashed yellow curve. **c.** Gp15 carboxyl terminus bound to the gp6 clip pocket of the connector. The last seven residues (95-102) of gp15 are rendered as surface (left) and sticks (right) in magenta. All views are shown from the tail side.

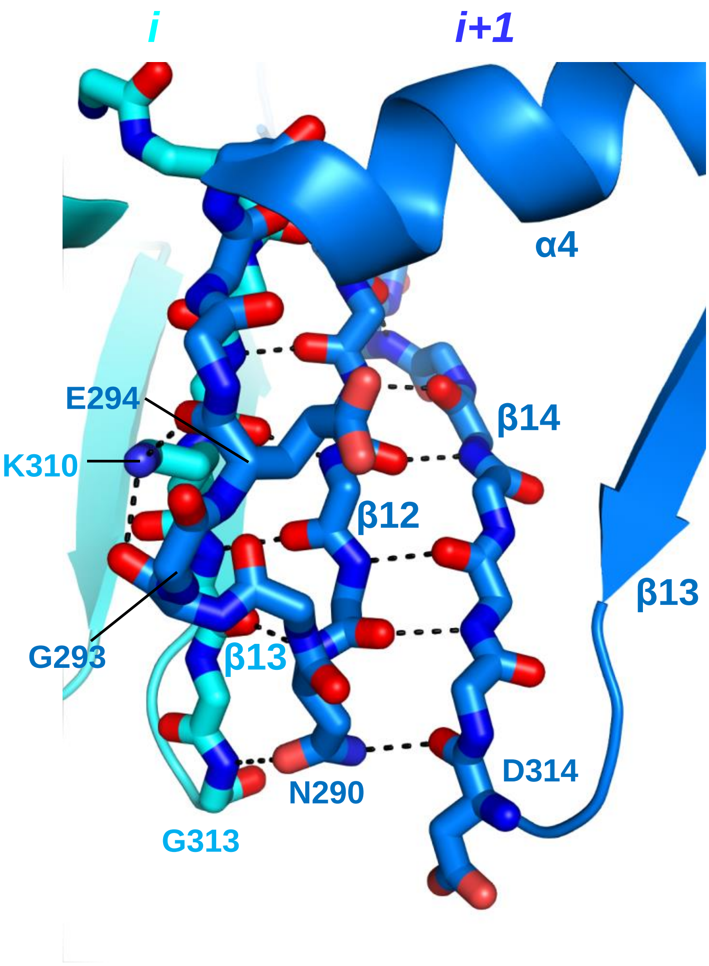

Supplementary Fig 4. Bonding network of gp6 clip residues N290, G293 and E294. Subunits *i* and *i+1* are coloured as in Fig. 1b. Bonds are shown by black dashed lines.

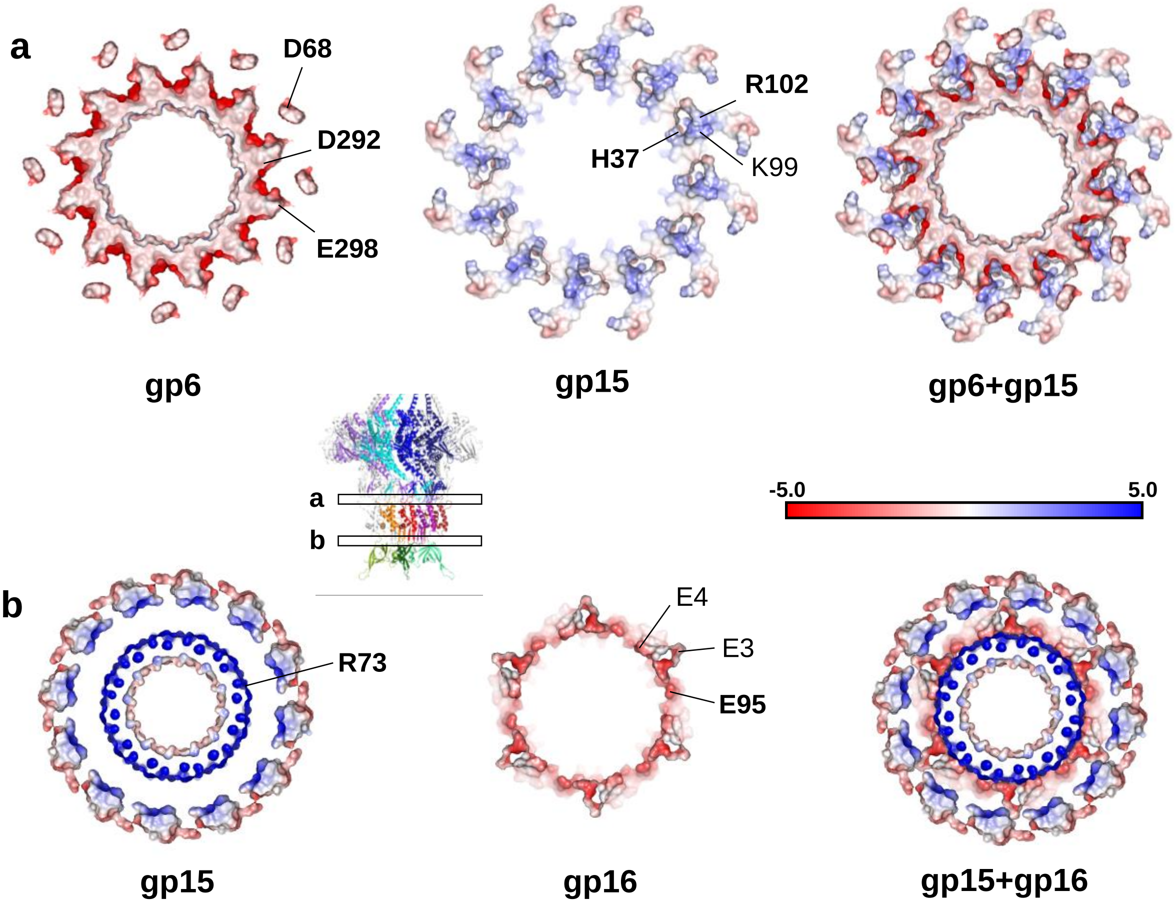

Supplementary Fig 5. Electrostatic potential at the gp6-gp15 and gp15-gp16 interfaces.

a. Slab of the local electrostatic potential of the gp6 clip bottom region (left) and its interacting gp15 interface (centre). The slab through the two proteins (right) highlights electrostatic potential complementarity and shape match. Electrostatic potentials are rendered in red and blue from -5.0 kT/e (red) to 5.0 kT/e (blue); see the scale bar in the middle of the figure on the right side. Residues establishing gp6-gp15 saline bridges are labelled in bold and charged residues are identified as well. **b.** Slab of the local electrostatic potential complementarity of the gp15 (left) and gp16 (centre) demonstrates interaction interfaces at the level of Arg73 in the β-barrel of gp15 (right panel). The positions of slabs in **a** and **b** are shown in the connector view in the centre of the figure. All views are shown from the tail side.

a

protein	phage	oligo state	structure	PDB	Z-score	Reference
**gp15**	siphophage SPP1	12	cryoEM	7Z4W		this work
**gp6**	siphophage HK97	13	X-rays	3JVO	9.2	2
**YqbG**	skin element *B subtilis*	1	NMR	1ZTS	6.4	3
**gp36**	myophage Mu	1	NMR	5YDN	6.4	4
**gp11**	podophage T7	12	cryoEM	6R21	5.3	5
**gp15**	siphophage SPP1	1	NMR	2KBZ	4.1	6
**Distant homologues:**						
**gp4**	podophage P22	12	X-rays	4V4K		7
**lower collar**	podophage P68	12	cryoEM	6IAC		8
**gp11**	podophage φ29	12	cryoEM	6QZF		9

b

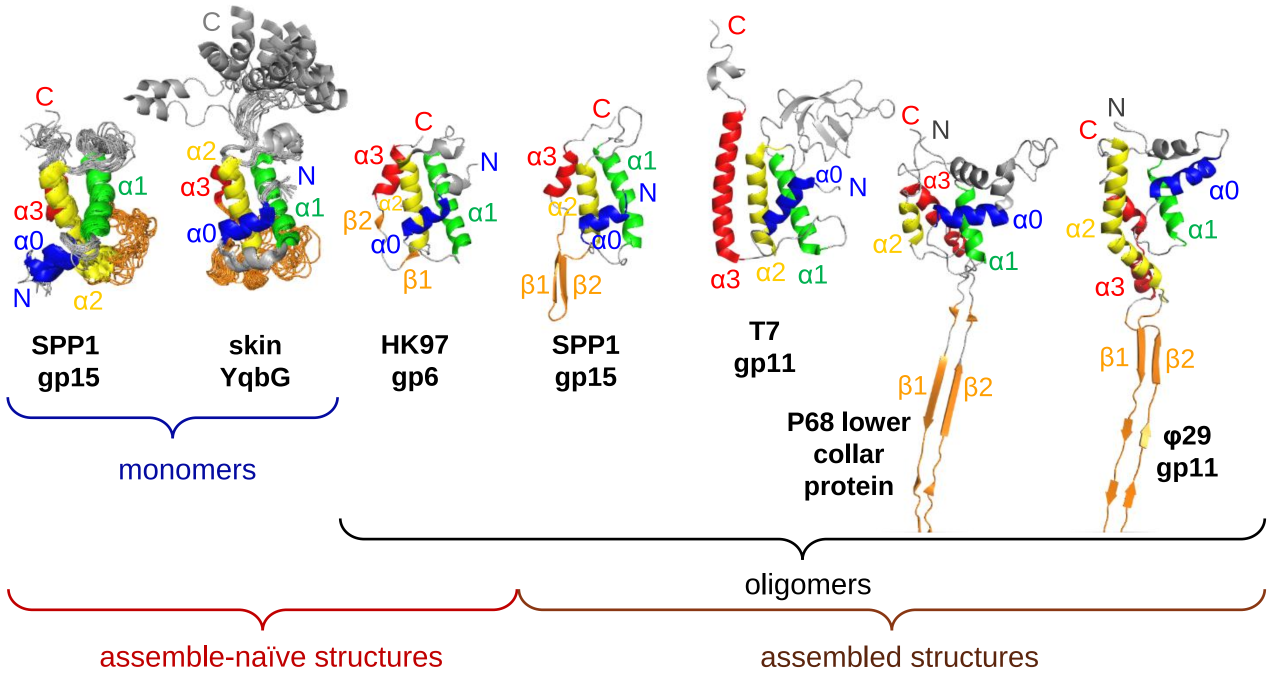

Supplementary Fig 6. Gp15 homologous proteins conformational landscape.

Supplementary Fig 6. Gp15 homologous proteins conformational landscape.

a. SPP1 gp15 structural homologues detected with DALI[1] (Z-scores are indicated) or identified by inspection of available structures of connector complexes in cases of more distant homologues. The state of oligomerisation, structure determination method and the PDB coordinates codes are shown in the table. **b.** Atomic models of monomers[2-9] (twenty superimposed NMR structures of SPP1 gp15 and of skin element YqbG), and of single subunits from oligomers of assembly-naïve (HK97 gp6) and assembled viral particles SPP1 gp15[6], T7 gp11[5], P68 lower collar protein[8] and φ29 gp11[9]. The structures are aligned with SPP1 gp15 of the connector through helices α1, α2 and α3. Their secondary structure elements are labeled according to the SPP1 gp15 structure and colored from blue (α0) to red (helix α3 and C-terminus). Structural elements that do not present in gp15 are shown in grey. The regions extending β1-β2 outwards from the protein core of phages P68 and φ29 are truncated.

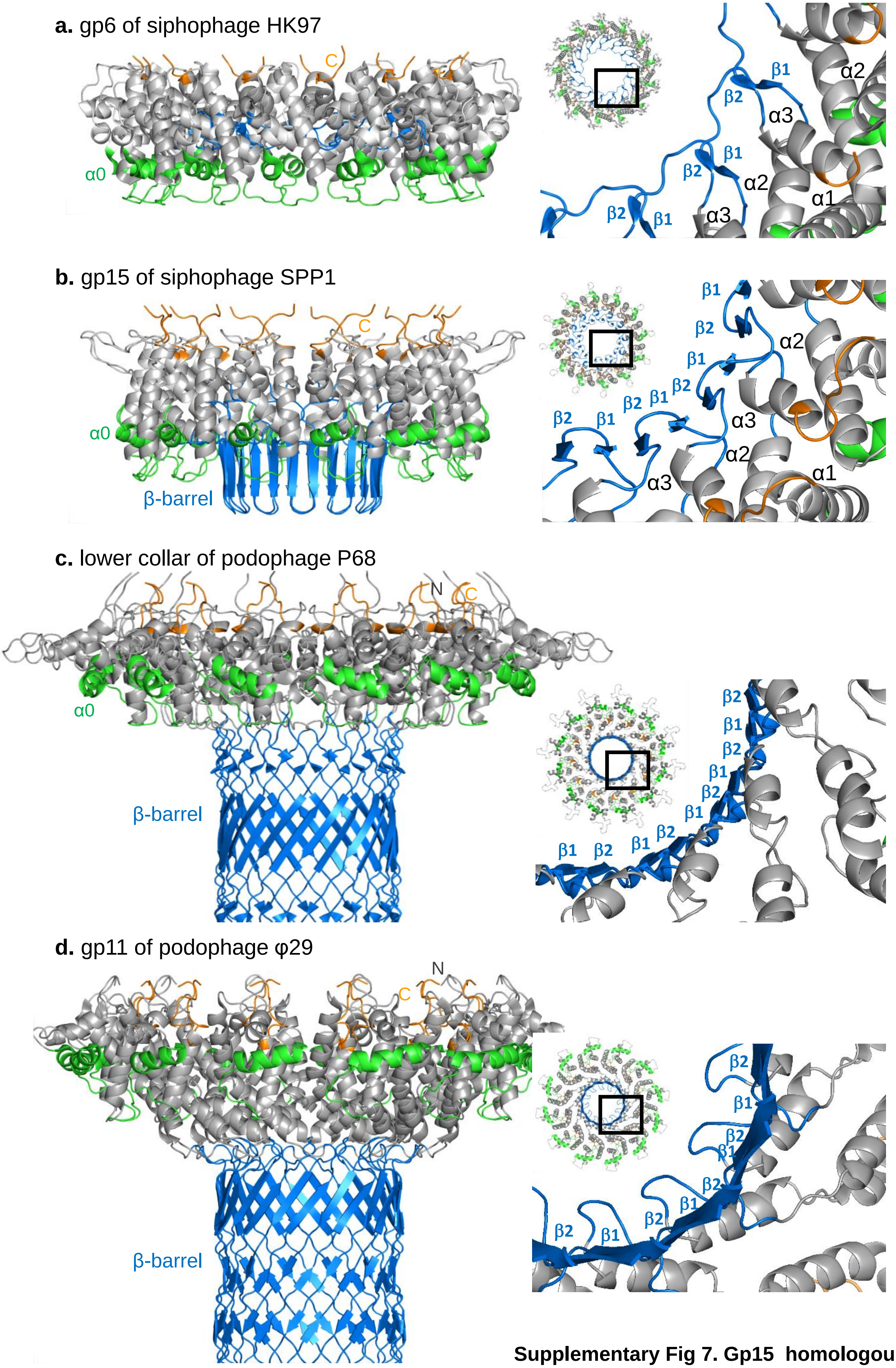

Supplementary Fig 7. Gp15 homologous oligomers.

Supplementary Fig 7. Gp15 homologous oligomers.

Left panels show side views of the oligomers; right panels demonstrate end views (from the capsid) accordingly. The position of zoomed in regions are identified by rectangles in the inserts. Equivalent structural elements are shown in similar colors. The flexible C-termini that anchor these proteins to the portal complexes are shown in orange, helices corresponding to α0 are in green, and the β-barrels formed upon folding of loop α2-α3 into the β1-β2 hairpin are in blue.

a. Siphophage HK97 assembly-naïve gp6 13-mer purified from an overproducing strain[2] (PDB 3JVO), showing an anti-parallel β-like interchain interaction at the position similar to the β-barrel of structures in **b-d**. **b.** Siphophage SPP1 gp15 structure in the connector. **c.** Podophage P68 lower collar protein structure in the phage particle[8] (PDB 6IAC). The β-barrel forms the short tail DNA conduit. Its bottom part is truncated in the figure. **d.** Podophage φ29 gp11 in the phage particle[9] (PDB 6QZF). In **c** and **d,** the β-barrel forms the short tail DNA conduit bottom parts of which are truncated in the figure.

a

protein	phage	oligo state	structure	PDB	Z-score	Reference
**gp16**	siphophage SPP1	6	cryoEM	7Z4W		this work
**SF1141**	protein from *Shigella flexneri* 2a	1	NMR	2KZ4	12.7	
**XkdH**	*B subtilis* phage-like element PBSX	1	Xray	3F3B	8.5	
**gp16**	siphophage SPP1	1	NMR	2KCA	6.1	6
**gpFII**	siphophage λ	1	NMR	1K0H	4.5	10
**distant homologues:**						
**gp9**	podophage φ29	6	cryoEM	6QZF		9

b

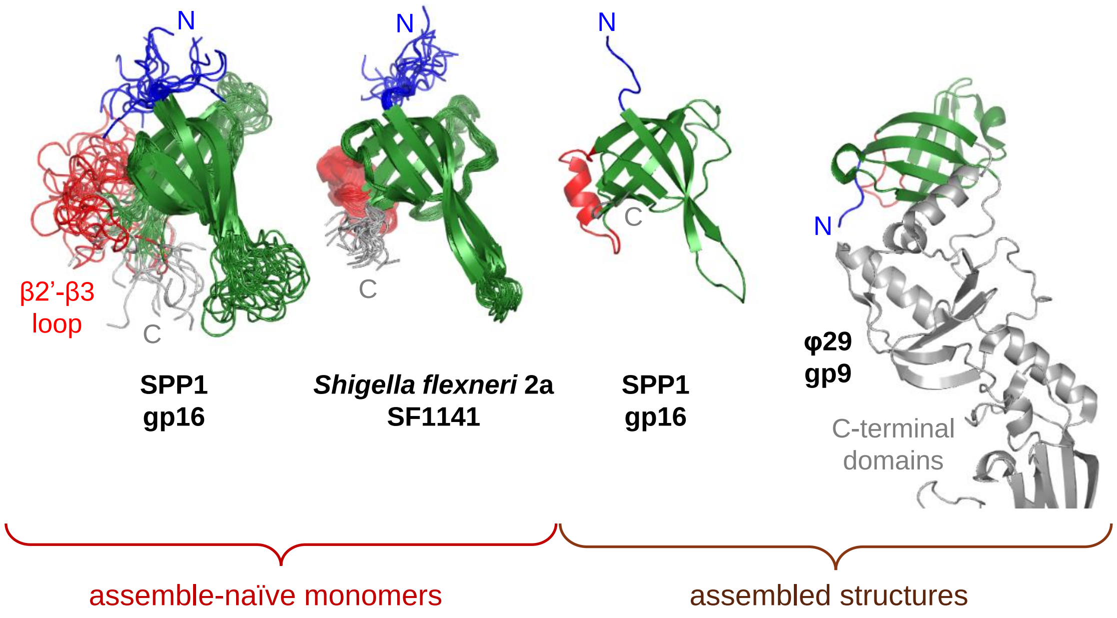

Supplementary Fig 8. Gp16 homologous proteins conformational landscape.

Supplementary Fig 8. Gp16 homologous proteins conformational landscape.

a. SPP1 gp16 structural homologues detected with DALI[1] (Z-score displayed) or identified by inspection of available phage particle (sub-)structures in case of the distantly related domain of the tail knob protein gp9 from phage φ29. The oligomerisation state, structure determination method and the PDB code are indicated in the table. **b.** Atomic models of monomers (twenty superimposed NMR structures of SPP1 gp15 and of *Shigella flexneri* 2a SF1141) and of subunits from oligomers of assembled SPP1 and φ29 viral particle complexes.The structures are aligned with SPP1 gp16 through the β-barrel core. Structural elements are colored as in Fig 4a. The N-terminal domain of φ29 gp9, which interacts with the β-barrel region of gp11 (Supplementary Fig 7d) distal from the capsid[9], is shown in grey.

Supplementary Table 1. Cryo-EM analysis of the SPP1 connector: data collection, refinement and validation statistics.

EMDB, PDB	EMD-14509, 7Z4W
Microscope (Voltage)	Titan Krios (300kV)
Pixel size (Å/pixel)	0.69
Camera	Falcon III
Defocus range (μm)	-1.2 to -2.5
Overall electron dose (e^-/ Å^2)	45
Symmetry	C6
Number of micrographs	3,876
Initial number of particles	524,494
Final number of particles	401,807
Map res. range (threshold 0.143)	2.27-3.0
Map Sharpening B Factor	-90
**Refinement**	
Initial Model (PDB)	*ab initio* modelling
Model Resolution (Å)	2.7
CC Model vs. Data (Mask)	0.83
**Model composition**	
Non-hydrogen atoms	17298
Protein residues	gp6 447; gp15 102; gp16 109
**RMSD**	
Bond Lengths	0.008
Bond angles	1.117
**Validation**	
Molprobity Score	1.21
Clashscore	1.86
**Ramachandran plot**	
Favoured (%)	96.19
Outliers (%)	0.08

Supplementary Table 2. Single amino acid substitutions in SPP1 gp6 impairing specifically viral DNA packaging.

Mutations are listed on the left lane. They are grouped according to their position on gp6 structural elements (Fig. 2e). The position and structural environment of the residue in the connector gp6 structure are presented together with a description of the mutation effect when interpretable. Their position and structural effects in elements of gp6 in the connector are presented. The phenotype associated to each mutation, previously reported[11-14], is also described. The phenotypes strength is based on results of a trans-complementation assay: very strong < 2 %; strong 2-5 %; moderate 5-10 % complementation.

Mutation	Group	Structural environment / Effect	Phenotype
**Tyr52Cys**	β-hairpin anchor	Buried, intrachain H-bonds to Asp58, Lys261, Asp265 / Local destabilization by mutation.	Very strong phenotype, no DNA packaging[11].
**Asp58Asn**		Exposed to the portal exterior, intrachain H-bonds to Tyr52, Lys261.	Strong phenotype, no DNA packaging[11].
**Asn82Asp**		Semi-buried, interchain bonds to Asn266 in helix α3 / Possibly positioning the β-hairpin relative to the stem.	Strong phenotype, no DNA packaging[11].
**His89Tyr**		Buried, intrachain H-bonds to His260, Asn139 / Local destabilization by mutation.	Very strong phenotype, no DNA packaging[11].
**Lys96Arg**		Buried, intrachain H-bonds to Asp256, Asn249, Ser138 / Local destabilization by mutation.	Strong phenotype, no DNA packaging[11].
**Gly153Arg**	wing	Exposed, intrachain main chain H-bonds to Tyr395, facing intrachain Lys232 and Gly233 main chain / Local destabilization by mutation.	Very strong phenotype, no DNA packaging[11].
**Gly153Ala**		Exposed, intrachain main chain H-bonds to Tyr395, facing Lys232 and Gly233 main chain / Local destabilization by mutation.	Strong phenotype, no DNA packaging[11].
**Ala180Thr**		Buried / Local destabilization.	Moderate phenotype, low DNA packaging efficiency[11].
**Glu198Leu**		Buried, intrachain salt bridge to Arg182, H-bond to Tyr200 / Local destabilization.	Strong phenotype, no DNA packaging[11].

Mutation	Group	Structural environment / Effect	Phenotype
**Asn290Thr**	clip interface	Exposed at the gp6 bottom, intrachain H-bond to Asp314(mc), interchain H-bond Gly313(mc), interchain main chain H-bond to Val311(mc). Interacts with gp15: main chain H-bond to gp15 Arg98 and side chain H-bond to gp15 Asn95.	Very strong phenotype, no DNA packaging[11]. No DNA packaging in vitro, no stimulation of gp2 ATPase activity[12] but interacts with gp2[13].
**Gly293Arg**	clip interface	Exposed at the gp6 bottom. Interacts with gp15: main chain H-bond to gp15 Arg98(mc).	Strong phenotype, low DNA packaging efficiency[11,14].
**Glu294Lys**	clip interface	Exposed at the gp6 bottom. Interchain main chain H-bond to Lys310(mc) / Mutation does not disrupt H-bond. Interacts with gp15: side chain bond to gp15 Met100 / Mutation might destabilize the gp15 interaction.	Very strong phenotype, no DNA packaging[11]. No stimulation of gp2 ATPase activity[12].
**Glu294Gly**	clip interface	Exposed at the gp6 bottom. Interchain main chain H-bond to Lys310(mc) / Mutation does not disrupt H-bond. Interacts with gp15: side chain bond to gp15 Met100 / Mutation disrupts this interaction.	Very strong phenotype[11]. Packaged DNA leaks from capsid. No interaction with gp15[14].
**Pro325Leu**	clip hinge	Hinge position of the clip. Interchain van der Waals contacts with Arg321, Glu323, Gln283, Ile284 / Mutation might affect clip position.	Strong phenotype, no DNA packaging[11].
**Val347Met**	tunnel loop	Buried, facing the bend of helix α6 / Local destabilization. Mutation might displace tunnel loop.	Moderate phenotype, no DNA packaging[11].
**Ser350Pro**	tunnel loop	Exposed to the tunnel in the tunnel loop. Intrachain side chain H-bond to Asp348 / Mutation disrupts this bond leading to a possible destabilization of the gp6 tunnel helix.	Strong phenotype, no DNA packaging[11].
**Glu352Gly**	tunnel loop	Exposed, residue in the tunnel loop helix / Mutation to glycine could affect the helix stability.	Moderate phenotype, low DNA packaging efficiency in vivo and in vitro[11,12]. Reduced stimulation of gp2 ATPase activity[12] but interacts with gp2[13].
**Gly360Val**	tunnel loop	Buried, at the N-ter of helix α6, intrachain H-bond to Glu424 / Substitution by a bulkier residue might affect position of helix α7 that lines the portal tunnel in the crown region.	Moderate phenotype, no DNA packaging[11,14].
**Lys373Glu**	tunnel loop	Buried, near the helix α6 distortion position. Side chain H-bond with the carbonyls of Ala344 and Gln345 / Mutation disrupts this interaction and may lead to displacement of the tunnel loop.	Strong phenotype, no DNA packaging[11].

Mutation	Group	Structural environment / Effect	Phenotype
**Ser428Gly**	crown	Buried, in the middle of helix α7, establishes helices α7-α7 intersubunit interface / Mutation by Gly could affect the conformation of α7 and could modify the interface with intersubunit α7.	Strong phenotype, no DNA packaging[11].
**Ser428Asn**		Buried, in the middle of helix α7, establishes helices α7-α7 intersubunit interface / Mutation could modify the interface with intersubunit α7	Moderate phenotype, no DNA packaging[11].
**Ile437Val**		Buried in a hydrophobic pocket build together with intersubunit residues Val426, Val430, Val433, Ala443, Val444, and hydrophobic section of Lys440, Asn447 of the neighbour subunit / Mutation disturbs crown intersubunit interactions	Moderate phenotype, no DNA packaging[11].
**Ala443Thr**		Buried in the hydrophobic core of the crown / Mutation probably disturbs the domain.	Strong phenotype, no DNA packaging[11]
**Phe449Leu**		Buried in a hydrophobic interface with Ile425, Leu429, Met438, Arg446 of the neighbour interface / Mutation disturbs crown intersubunit interactions.	Strong phenotype, no DNA packaging[11].

Supplementary Table 3. Intersubunit contacts in the SPP1 connector complex gp6 - gp15 bonding

gp6 residue location	gp6 residue (chain), atom	gp15 residue (chain), atom	gp15 residue location	type of interaction
β1	D68 (i+2), O	R102 (j+1), Nh1	C-ter	hydrogen bond
clip region	N290 (i), O	R98 (j+1), Ne	C-ter	hydrogen bond
	N290 (i), Od1	N95 (j+1), Nd2	α3	hydrogen bond
	D292 (i), N	R98 (j+1), O	C-ter	hydrogen bond
	D292 (i+1), Od2	H37 (j+1), Ne2	α1-α2 loop	salt bridge
	G293 (i), N	R98 (j+1), O	C-ter	hydrogen bond
	E294 (i), Oe1	M100 (j+1), N	C-ter	hydrogen bond
	E298 (i), Oe1	R102 (j+1), Nh1	C-ter	salt bridge
	E298 (i), Oe1	R102 (j+1), Nh2	C-ter	salt bridge
	K310 (i), Nz	R102 (j+1), O	C-ter	hydrogen bond
	S312 (i), O	R98 (j+1), Nh2	C-ter	hydrogen bond

Supplementary Table 3 (cont.) Intersubunit contacts in the SPP1 connector complex

gp15 - gp15 intersubunit bonding

gp15 chain j residue location	gp15 chain j residue, atom	gp15 chain j-1 residue, atom	gp15 chain j-1 residue location	type of interaction
α0	R5, Ne	E23, Oe1	α1	salt bridge
α0	R8, Ne	E23, Oe2	α1	salt bridge
α0	R8, Nh2	E23, Oe2	α1	salt bridge
α2	S51, Og	D34, Od2	α1	hydrogen bond
α2	K59, Nz	Y64, O	α2	hydrogen bond
α2	K59, Nz	T66, O	α2-β1 loop	hydrogen bond
α2	Q62, Oe1	A68, N	α2-β1 loop	hydrogen bond
β1-β2 loop	T77, O	D76, N	β1-β2 loop	hydrogen bond
β2	S79, N	S74, O	β1	hydrogen bond
β2	S79, O	S74, N	β1	hydrogen bond
β2	N81, N	G72, O	β1	hydrogen bond
β2	N81, O	T71, N	β1	hydrogen bond
β2	N81, O	T71, Og1	β1	hydrogen bond
β2	N81, O	G72, N	β1	hydrogen bond
β2-α3 loop	A83, N	G69, O	α2-β1 loop	hydrogen bond
α3	S88, N	E85, Oe1	β2-α3 loop	hydrogen bond
α3	S88, N	E85, Oe2	β2-α3 loop	hydrogen bond
α3	S88, Og	E85, Oe1	β2-α3 loop	hydrogen bond
α3	S88, Og	E85, Oe2	β2-α3 loop	hydrogen bond
α3	T89, N	E85, Oe1	β2-α3 loop	hydrogen bond
α3	T89, Og1	E85, Oe1	β2-α3 loop	hydrogen bond
α3	T89, Og1	I86, N	β2-α3 loop	hydrogen bond
C-ter	Y97, Oh	D34, Od2	α1	hydrogen bond

Supplementary Table 3 (cont.) Intersubunit contacts in the SPP1 connector complex

gp15 - gp16 bonding

gp15 residue location	gp15 residue (chain), atom	gp16 chain k residue, atom	gp16 chain k residue location	type of interaction
α2	M65 (j-1), Sd	M1, N	N-ter	hydrogen bond
β1	R73 (j-1), Nh1	Y2, O	N-ter	hydrogen bond
β2	Y80 (j), Oh	E4, Oe1	N-ter	hydrogen bond
β1	M75 (j-1), O	Q39, Ne2	β2’	hydrogen bond
β1-β2 loop	T77 (j-1), N	Q39, Oe1	β2’	hydrogen bond
β1-β2 loop	T77 (j-1), Og1	Q39, Oe1	β2’	hydrogen bond
β1-β2 loop	D76 (j-2), Od1	Q43, N	α1	hydrogen bond
β1-β2 loop	D76 (j-2), Od2	Q43, N	α1	hydrogen bond
β1-β2 loop	D76 (j-1), Od2	N59, Nd2	β3	hydrogen bond
β1-β2 loop	T77 (j), Og1	Y61, Oh	β3	hydrogen bond
β1-β2 loop	T77 (j+1), Og1	S91, Og	β5-β6 loop	hydrogen bond
β1	R73 (j), Nh1	E95, Oe1	β5-β6 loop	salt bridge
β1	R73 (j), Nh2	E95, Oe1	β5-β6 loop	salt bridge
β1-β2 loop	D76 (j), Od2	R98, Nh1	β6	salt bridge
β1-β2 loop	T77 (j), Og1	R98, Nh1	β6	hydrogen bond
β1-β2 loop	D76 (j), Od2	R98, Nh2	β6	salt bridge

gp16 - gp16 intersubunit bonding

gp16 chain k residue location	gp16 chain k residue, atom	gp16 chain k-1 residue, atom	gp16 chain k-1 Residue location	type of interaction
β2’	H37, Ne2	S91, O	β5-β6 loop	hydrogen bond
α1	Q43, Ne2	E45, Oe2	α1	hydrogen bond
	Q43, Ne2	N59, Od1	β3	hydrogen bond
	Q43, Oe1	Y61, Oh	β3	hydrogen bond
	Q43, Oe1	R98, Nh1	β6	hydrogen bond
	Y47, Oh	E45, Oe2	α1	hydrogen bond
β4-β5 loop	R77, Nh1	G92, O	β5-β6 loop	hydrogen bond

Supplementary Table 4. Surface, energetics and bonding of connector interchain interfaces

	subunits interface	subunit 1 (chain)	subunit 2 (chain)	interface area (Å²)	ΔG (kcal/mol)	Nb of H-bonds	Nb of salt bridges	total interface area (Å²)	total BSA* (Å²)
**gp6 (*i*)**	gp6/gp6	gp6 (*i*)	gp6 (*i-2*)	374.4	-3.4	4	0	8486.7	9192.3
		gp6 (*i*)	gp6 (*i-1*)	3878.8	-40.2	43	11		
		gp6 (*i*)	gp6 (*i+1*)	3859.2	-39.9	43	11		
		gp6 (*i*)	gp6 (*i+2*)	374.3	-3.4	4	0		
	gp6/gp15	gp6 (*i*)	gp15 (*j-1*)	79.9	-0.2	1	0	705.6	
		gp6 (*i*)	gp15 (*j*)	82.1	-0.8	0	1		
		gp6 (*i*)	gp15 (*j+1*)	516.0	-0.6	8	3		
		gp6 (*i*)	gp15 (*j+2*)	27.6	0.0	0	0		
**gp15 (*j-1*)**	gp15/gp6	gp15 (*j-1*)	gp6 (*i-3*)	26.8	0.0	1	0	702.9	3779.3
		gp15 (*j-1*)	gp6 (*i-2*)	516.4	-0.6	7	2		
		gp15 (*j-1*)	gp6 (*i-1*)	79.8	-0.8	0	1		
		gp15 (*j-1*)	gp6 (*i*)	79.9	-0.2	1	0		
	gp15/gp15	gp15 (*j-1*)	gp15 (*j-2*)	1244.6	-13.2	20	3	2503.6	
		gp15 (*j-1*)	gp15 (*j*)	1259.0	-13.3	20	3		
	gp15/gp16	gp15 (*j-1*)	gp16 (*k-1*)	66.5	-0.7	1	0	572.3	
		gp15 (*j-1*)	gp16 (*k*)	480.3	-7.6	6	0		
		gp15 (*j-1*)	gp16 (*k+1*)	26.0	0.5	0	0		
**gp15 (*j*)**	gp15/gp6	gp15 (*j*)	gp6 (*i-2*)	27.9	0.0	1	0	700.2	3657.0
		gp15 (*j*)	gp6 (*i-1*)	512.7	0.1	7	2		
		gp15 (*j*)	gp6 (*i*)	82.1	-0.8	0	1		
		gp15 (*j*)	gp6 (*i+1*)	77.5	-0.2	1	0		
	gp15/gp15	gp15 (*j*)	gp15 (*j-1*)	1254.4	-13.2	20	3	2491.3	
		gp15 (*j*)	gp15 (*j+1*)	1236.9	-13.1	20	3		
	gp15/gp16	gp15 (*j*)	gp16 (*k*)	318.5	-2.8	4	3	465.5	
		gp15 (*j*)	gp16 (*k+1*)	147.0	-0.8	0	0		
**gp16 (*k*)**	gp16/gp15	gp16 (*k*)	gp15 (*j-3*)	26.6	0.6	0	0	1040.8	2060.6
		gp16 (*k*)	gp15 (*j-2*)	147.9	-0.8	2	0		
		gp16 (*k*)	gp15 (*j-1*)	480.3	-7.6	6	0		
		gp16 (*k*)	gp15 (*j*)	318.5	-2.8	3	4		
		gp16 (*k*)	gp15 (*j+1*)	67.5	-0.7	2	0		
	gp16/gp16	gp16 (*k*)	gp16 (*k-1*)	509.8	-1.9	7	0	1019.8	
		gp16 (*k*)	gp16 (*k+1*)	510.0	-2.0	7	0		

*BSA : Buried Surface Area

Supplementary References

1. Holm, L. DALI and the persistence of protein shape. *Protein Sci.* **29**, 128–140 (2020).
2. Cardarelli, L. *et al.* The Crystal Structure of Bacteriophage HK97 gp6: Defining a Large Family of Head–Tail Connector Proteins. *J. Mol. Biol.* **395**, 754–768 (2010).
3. Liu, G. *et al.* NMR structure of protein yqbG from Bacillus subtilis reveals a novel α-helical protein fold. *Proteins Struct. Funct. Bioinforma.* **62**, 288–291 (2005).
4. Iwasaki, T. *et al.* Three-dimensional structures of bacteriophage neck subunits are shared in Podoviridae , Siphoviridae and Myoviridae. *Genes to Cells* **23**, 528–536 (2018).
5. Cuervo, A. *et al.* Structures of T7 bacteriophage portal and tail suggest a viral DNA retention and ejection mechanism. *Nat. Commun.* **10**, 3746 (2019).
6. Lhuillier, S. *et al.* Structure of bacteriophage SPP1 head-to-tail connection reveals mechanism for viral DNA gating. *Proc. Natl. Acad. Sci. U. S. A.* **106**, 8507–8512 (2009).
7. Olia, A. S., Prevelige, P. E., Johnson, J. E. & Cingolani, G. Three-dimensional structure of a viral genome-delivery portal vertex. *Nat. Struct. Mol. Biol.* **18**, 597–603 (2011).
8. Hrebík, D. *et al.* Structure and genome ejection mechanism of Staphylococcus aureus phage P68. *Sci. Adv.* **5**, (2019).
9. Xu, J., Wang, D., Gui, M. & Xiang, Y. Structural assembly of the tailed bacteriophage ϕ29. *Nat. Commun.* **10**, 2366 (2019).
10. Maxwell, K. L., Yee, A. A., Arrowsmith, C. H., Gold, M. & Davidson, A. R. The Solution Structure of the Bacteriophage λ Head–Tail Joining Protein, gpFII. *J. Mol. Biol.* **318**, 1395–1404 (2002).
11. Isidro, A., Santos, M. A., Henriques, A. O. & Tavares, P. The high-resolution functional map of bacteriophage SPP1 portal protein. *Mol. Microbiol.* **51**, 949–962 (2004).
12. Oliveira, L., Henriques, A. O. & Tavares, P. Modulation of the viral ATPase activity by the portal protein correlates with DNA packaging efficiency. *J. Biol. Chem.* **281**, 21914–21923 (2006).
13. Oliveira, L., Cuervo, A. & Tavares, P. Direct Interaction of the Bacteriophage SPP1 Packaging ATPase with the Portal Protein. *J. Biol. Chem.* **285**, 7366–7373 (2010).
14. Isidro, A., Henriques, A. O. & Tavares, P. The portal protein plays essential roles at different steps of the SPP1 DNA packaging process. *Virology* **322**, 253–263 (2004).
