# Supplementary material for: CryoEM structure and assembly mechanism of a bacterial virus genome gatekeeper
Source: Nat Commun. 2022 Nov 26;13:7283. doi: 10.1038/s41467-022-34999-8 (PMC9701221; doi:10.1038/s41467-022-34999-8)
Supplement: Supplementary file 3 — Description of Additional Supplementary Files [file 41467_2022_34999_MOESM3_ESM.pdf]

File name: Supplementary Movie 1

Description: Bacteriophage SPP1 connector assembly mechanism.

SPP1, intersubunit bonding of α0 that bridges helices α1 of neighbor subunits. Re-positioning of α0 is thus an essential assembly step. Changes in loops α2-α3 of adjacent subunits in gp15 dictate conformational changes in their fold. These changes result conceivably in formation of the β-barrel during gp15 oligomerization.
