# Supplementary material for: CryoEM structure and assembly mechanism of a bacterial virus genome gatekeeper
Source: Nat Commun. 2022 Nov 26;13:7283. doi: 10.1038/s41467-022-34999-8 (PMC9701221; doi:10.1038/s41467-022-34999-8)
Supplement: Supplementary file 5 — Reporting Summary [file 41467_2022_34999_MOESM5_ESM.pdf]

nature portfolio

Corresponding author(s): E.V. Orlova

Last updated by author(s): 7 Nov_2022

Reporting Summary

Nature Portfolio wishes to improve the reproducibility of the work that we publish. This form provides structure for consistency and transparency in reporting. For further information on Nature Portfolio policies, see our Editorial Policies and the Editorial Policy Checklist.

Statistics

For all statistical analyses, confirm that the following items are present in the figure legend, table legend, main text, or Methods section.

n/a	Confirmed	
☐	☒	The exact sample size (*n*) for each experimental group/condition, given as a discrete number and unit of measurement
☒	☐	A statement on whether measurements were taken from distinct samples or whether the same sample was measured repeatedly
☒	☐	The statistical test(s) used AND whether they are one- or two-sided<br>*Only common tests should be described solely by name; describe more complex techniques in the Methods section.*
☒	☐	A description of all covariates tested
☒	☐	A description of any assumptions or corrections, such as tests of normality and adjustment for multiple comparisons
☒	☐	A full description of the statistical parameters including central tendency (e.g. means) or other basic estimates (e.g. regression coefficient) AND variation (e.g. standard deviation) or associated estimates of uncertainty (e.g. confidence intervals)
☒	☐	For null hypothesis testing, the test statistic (e.g. *F*, *t*, *r*) with confidence intervals, effect sizes, degrees of freedom and *P* value noted<br>*Give P values as exact values whenever suitable.*
☒	☐	For Bayesian analysis, information on the choice of priors and Markov chain Monte Carlo settings
☒	☐	For hierarchical and complex designs, identification of the appropriate level for tests and full reporting of outcomes
☒	☐	Estimates of effect sizes (e.g. Cohen's *d*, Pearson's *r*), indicating how they were calculated

Our web collection on statistics for biologists contains articles on many of the points above.

Software and code

Policy information about availability of computer code

Data collection	Data were collected on a Titan Krios microscope (ThermoFisherTM, the eBIC Diamond light source facility, Harwell, Oxfordshire, UK) operating at 300 kV with the specimen maintained at liquid nitrogen temperatures. Images were recorded using a Falcon III camera (ThermoFisherTM) in integrating mode that enables faster data acquisition. Data collection was done using the EPU software (ThermoFisherTM) within a defocus range of -1.2 to -2.5 µm. Movies (40 frames per movie) were collected with a dose of 1.12 e-/Å2 per frame at the specimen plane over a 1 s exposure. The calibrated pixel size was 0.69 Å at the specimen level.
Data analysis	MotionCorr2; CTFFIND4; Relion 2.0; CryoSPARC-2; Coot 0.8.9.1; Phenix 1.19-4092; DaliLitev5 (http://ekhidna2.biocenter.helsinki.fi/dali/), Chimera 1.16 (UCSF Resource for Biocomputing, Visualization, and Informatics)

For manuscripts utilizing custom algorithms or software that are central to the research but not yet described in published literature, software must be made available to editors and reviewers. We strongly encourage code deposition in a community repository (e.g. GitHub). See the Nature Portfolio guidelines for submitting code & software for further information.

Data

Policy information about availability of data

All manuscripts must include a data availability statement. This statement should provide the following information, where applicable:

- Accession codes, unique identifiers, or web links for publicly available datasets
- A description of any restrictions on data availability
- For clinical datasets or third party data, please ensure that the statement adheres to our policy

The data that support this study are available from the corresponding authors upon reasonable request. Coordinates and structure factor amplitudes have been deposited in the Protein Data Bank under the accession code 7Z4W [and the corresponding electron microscopy density map has been deposited in the Electron Microscopy Data Bank under the accession code EMD-14509 [https://www.ebi.ac.uk/emdb/EMD-14509] (gp612 - gp1512 - gp166 complex).

Human research participants

Policy information about studies involving human research participants and Sex and Gender in Research.

Reporting on sex and gender	n/a
Population characteristics	n/a
Recruitment	n/a
Ethics oversight	n/a

Note that full information on the approval of the study protocol must also be provided in the manuscript.

Field-specific reporting

Please select the one below that is the best fit for your research. If you are not sure, read the appropriate sections before making your selection.

☒ Life sciences ☐ Behavioural & social sciences ☐ Ecological, evolutionary & environmental sciences

For a reference copy of the document with all sections, see nature.com/documents/nr-reporting-summary-flat.pdf

Life sciences study design

All studies must disclose on these points even when the disclosure is negative.

Sample size	Sample sizes were assessed by mesuraments of obtained 3D map of densities, based on the pixel size during data collection
Data exclusions	All data set was processed to reveal images of the paricles that were distorted , analysis was done using two-dimentional statistical classification in Relion and CryoSPARC
Replication	Replication experiments were not used
Randomization	Statistical analysis of particle images was based on K-means method used for 2D classification in Relion
Blinding	This approach was not used in this study.

Reporting for specific materials, systems and methods

We require information from authors about some types of materials, experimental systems and methods used in many studies. Here, indicate whether each material, system or method listed is relevant to your study. If you are not sure if a list item applies to your research, read the appropriate section before selecting a response.

Materials & experimental systems

n/a	Involved in the study	
☒	☐	Antibodies
☒	☐	Eukaryotic cell lines
☒	☐	Palaeontology and archaeology
☒	☐	Animals and other organisms
☒	☐	Clinical data
☒	☐	Dual use research of concern

Methods

n/a	Involved in the study	
☒	☐	ChIP-seq
☒	☐	Flow cytometry
☒	☐	MRI-based neuroimaging
